# Missões religiosas e património africano nos museus etnográficos: a circulação de umacoleção missionária reunida em Angola,entre o colonial e o pós-colonial

**DOI:** 10.1590/S0104-59702025000100029

**Published:** 2025-08-18

**Authors:** Ana Rita Amaral

**Affiliations:** i Pesquisadora de pós-doutorado, Universidade de Utrecht. Utrecht – Países Baixos ana.r.amaral@gmail.com

**Keywords:** Coleções museais, Proveniência, Antropologia, Legados coloniais e missionários, Património angolano, Museum collections, Provenance, Anthropology, Colonial and missionary legacies, Angolan heritage

## Abstract

Este artigo aborda uma camada da história de uma coleção reunida pelos missionários da Congregação do Espírito Santo durante o período colonial em Angola. Essa coleção constituiu um museu missionário nas décadas de 1950 e 1960, sendo transferida, já depois do 25 de abril e da independência de Angola, para o então Museu e Laboratório Antropológico da Universidade de Coimbra, atual Museu da Ciência da Universidade de Coimbra. Revisita-se a pesquisa realizada sobre a coleção, considerando algumas questões levantadas em projetos recentes dedicados à “pesquisa de proveniência” de coleções coloniais, e abre-se a discussão sobre as coleções associadas à missionação como legados situados na intersecção entre religião, colonialismo e património.

## Abstract (en)

This article addresses one layer of the history of a collection gathered by missionaries from the Congregação do Espirito Santo during the colonial period in Angola. This collection comprised a missionary museum in the 1950s and 1960s, and after the April 25 Revolution and Angola’s independence was transferred to the Museum and Anthropology Laboratory at the University of Coimbra (now known as the Science Museum at the University of Coimbra). We reexamine research on this collection, considering some issues raised in recent projects dedicated to provenance studies on colonial collections, and discuss collections associated with religious missions as legacies where religion, colonialism and heritage intersect.

## Abordagens à história das coleções coloniais nos museus

As duas últimas décadas têm sido especialmente férteis em debates e estudos sobre a história das coleções museais reunidas durante o colonialismo e hoje contidas em vários museus europeus. A questão do seu estatuto e futuro, incluindo a possibilidade de restituição, tem sido objeto de grande atenção. Igualmente se tem assistido a um processo de transformação dos próprios museus, em particular daqueles que têm vindo a perder as adjetivações de “etnológicos”, “etnográficos” ou “antropológicos”. Essas mudanças têm sido impulsionadas por debates públicos mais abrangentes em torno da relação complexa e problemática entre museus e colonialismo, comportando implicações na maneira de conceber, gerir, expor e valorizar as coleções.

O estudo das coleções sempre constituiu uma função central desse tipo de museu, evocada, aliás, pelos qualificativos que vão sendo descartados gradualmente. Ou seja, os museus que, a dado momento, passaram a ser chamados “etnológicos” ou “antropológicos” eram assim concebidos como lugares onde se produzia e disseminava conhecimento a partir de coleções, segundo paradigmas que variaram ao longo do tempo, consoante a tradição disciplinar e as relações estabelecidas com as dinâmicas da ocupação colonial e da governação das populações colonizadas (a bibliografia é extensa; ver, por exemplo, Bouquet, 2001, 2012; Kuper, 2023; L’Estoile, 2007). O que se sabia sobre as coleções determinava, de maneiras diversas, não só o seu valor, mas o que se podia saber. A atribuição de classificações étnicas aos objetos e a verificação da sua “autenticidade” cultural, duas das principais tarefas desse tipo de museu, gerava necessariamente um interesse na sua trajetória. Por sua vez, a história das práticas de coleção e das coleções tem também sido alvo de estudos académicos desde, pelo menos, a década de 1990 (ver, por exemplo, Elsner, Cardinal, 1994; Findlen, 1994; Gosden, Knowles, 2001; Shelton, 2001a, 2001b; Stocking Jr., 1985; Thomas, 1989). Não obstante, as coleções e as suas histórias estão hoje no centro de uma atenção e de uma mobilização de fundos a uma escala sem precedentes, visando não só renovar as estruturas museais como promover pesquisas que respondam ao questionamento da legalidade e legitimidade da propriedade dessas mesmas coleções.

Só nos primeiros meses de 2024, por exemplo, era possível ler na imprensa artigos sobre a criação de um fundo de 2,1 milhões de euros, partilhado entre a França e a Alemanha, para promover a “pesquisa de proveniência de objetos patrimoniais africanos nas coleções dos seus museus nacionais, que possa preparar o seu eventual retorno” (Oltermann, 19 jan. 2024);^1^ sobre a abertura de uma nova exposição no recentemente remodelado Africa Museum, em Tervuren, na Bélgica, dedicada a “repensar coleções” (Vanhove, 21 jan. 2024); sobre a criação do “Consórcio Coleções Coloniais” pelo governo neerlandês, englobando várias instituições e com vista a promover a “pesquisa de proveniência” das coleções e a sua visibilidade e acessibilidade (Ondersteuning…, 29 fev. 2024); ou ainda sobre a criação do cargo de diretor de Pesquisa de Proveniência no Metropolitan Museum of Art para lidar com os crescentes escrutínios público, académico e mesmo policial relativamente à existência de objetos pilhados no acervo do conhecido museu nova-iorquino, assumindo o cargo um antigo profissional da leiloeira Sotheby’s (Pogrebin, 22 mar. 2024).

Não se pretende desenvolver aqui um levantamento exaustivo dessas iniciativas (ver, nesse sentido, Van Beurden, Gondola, Lacaille, 2023a). O que elas parecem trazer de novo, para além da escala, é o potencial inerente ao cruzamento de perspetivas e métodos de pesquisa entre os meios museal e académico, e entre estes e os seus interlocutores e públicos. Ou seja, por um lado, as mais diversas práticas museais são tomadas como base de problematizações mais gerais sobre as condições de produção e reprodução do conhecimento, sobre o estatuto epistémico e patrimonial das coleções e sobre o propósito dos museus. Por outro lado, as questões problematizadas pela pesquisa passaram a desestabilizar as práticas museais, nomeadamente perturbando critérios de classificação, conservação e valorização das coleções. Isso tem-se refletido, por exemplo, na revisão da nomenclatura utilizada nos catálogos e na mudança na tónica dos registos de aquisição. Estes deixam de responder apenas a questões de “autenticidade” e a auditorias de propriedade focadas exclusivamente na transação envolvendo o museu. Sublinham-se novos interesses em (1) tornar visíveis as circunstâncias históricas (muitas vezes violentas, outras vezes insólitas) que determinaram a maneira como as coleções foram obtidas, e (2) em abrir novas possibilidades de circulação. Essas dinâmicas comportam consequências institucionais, sociais e políticas mais vastas, nomeadamente no que respeita à formulação de novos enquadramentos normativos para as coleções e a formas mais criativas de envolvimento com elas, por exemplo, por meio de projetos colaborativos com partes interessadas nos atuais países associados à circulação das coleções e respetivas diásporas (ver, por exemplo, Golding, Modest, 2013; Von Oswald, Rodatus, 2017; Weber-Sinn, Ivanov, 2020).

Esse cruzamento potencial nem sempre é plenamente realizado, já que as abordagens desenvolvidas como “pesquisa de proveniência” por vezes seguem um ritmo acelerado e uma lógica forense, focada em traçar cadeias de propriedade e em detetar momentos singulares de aquisição para dar respostas operacionais à questão da restituição. É precisamente nesse sentido que se pode entender o apelo de Larissa Förster (2016) à necessidade de sistematizar e institucionalizar a “pesquisa de proveniência”, tomando-a como um fim em si, desacoplado da questão da restituição. No fundo, trata-se de sublinhar a relevância da pesquisa sobre a história e sobre o sentido das coleções nas práticas museais e académicas, o que não deixa de ir ao encontro do que já se vem fazendo há décadas, como anteriormente se referiu. No seu esforço de promover a “pesquisa de proveniência” nos museus do século XXI a uma historiografia mais abrangente e aprofundada das coleções, Förster (2016, p.51) alerta que ela “não se deve reduzir à mera associação de anedotas interessantes a certos objetos ou conjuntos de objetos”. Essa é uma observação crítica e interessante porque é reveladora de certa tendência factualista inerente às abordagens ancoradas na “proveniência”, que trata as informações constantes dos arquivos e bases de dados museais como “factos”, e não considera a historicidade precisamente das práticas de associar informações e narrativas aos objetos e às coleções.

Considerando essas questões mais recentes, mas tomando como base as discussões sobre o carácter instável e contingente do valor e do significado dos objetos nos museus (Handler, 1992; Shelton, 2000), a abordagem aqui adotada presta atenção às articulações e desarticulações da circulação de objetos e informação afetando, por essa via, o modo como aqueles são valorizados e (re)significados. Trata-se de examinar o que diversos autores têm designado como “trabalho categorial” (Bowker, Star, 2000), “trabalho historiográfico” (Roque, 2012) ou práticas de “catalogação da cultura” (Turner, 2020), ou seja, examinar que tipo de informações e narrativas se tornaram relevantes ao longo da trajetória dos objetos, bem como o modo como essas se expressam materialmente e os efeitos que comportam, antes e depois da sua incorporação museal. A partir daí, será aberta no final uma discussão sobre os legados dessas práticas para a maneira como as coleções são concebidas, descritas, valorizadas e circuladas hoje.

O artigo aborda, assim, a circulação histórica e epistémica de uma coleção reunida pelos missionários da Congregação do Espírito Santo em Angola durante o período colonial. Composta por quase um milhar de registos numerados, a coleção encontra-se hoje em depósito na secção de antropologia do Museu da Ciência da Universidade de Coimbra.^2^ Antes da sua transferência para esse museu, ocorrida já depois da independência de Angola e do fim do colonialismo português, a coleção fazia parte de um museu missionário criado pela Congregação num dos seus seminários em Portugal. Tal museu missionário foi constituído no início dos anos 1950, reunindo não só alguns dos objetos já existentes nas casas da Congregação, incluindo os que tinham sido exibidos na “Exposição de arte sacra missionária”, ocorrida em Lisboa em 1951, como também objetos trazidos de Angola pelos missionários, *grosso modo* entre as décadas de 1950 e 1960 (Amaral, 2018, 2020).

A análise segue a escala da coleção, e não a da trajetória singular de objetos. Ela foca-se nos contextos que condicionaram e nas narrativas que acompanharam a circulação da coleção já musealizada pelos missionários entre o seminário e o museu antropológico em Coimbra. Assim, segue-se uma estratégia conhecida nos estudos literários como *in medias res*, ou seja, começa-se pelo meio da história da coleção, pelo contexto que iria moldar a sua valorização e integração no então Museu e Laboratório Antropológico da Universidade de Coimbra. Trata-se de um momento situado entre o período colonial tardio, entre os anos 1950 e 1960, que viu ressurgir um interesse pelas coleções etnográficas em Coimbra, ancorado na sua condição “ultramarina”, e o período pós-colonial, que impôs um questionamento dessa condição e a sua revalorização como património africano a ser protegido em Portugal. Compreender essa dinâmica foi fundamental para o estudo da coleção tal como se encontra hoje, e que a seguir se apresenta. Este estudo procurou dar respostas claras para perguntas que, como alguns autores têm notado (Vanhee, 2023), os museus têm uma dificuldade inesperada em responder, tais como: qual a dimensão da coleção? Como descrever a sua composição? O que fez com que ela fosse parar em Coimbra? Como foram valorizados no museu antropológico objetos circulados no âmbito da evangelização católica em Angola? A dificuldade de responder a essas perguntas reside precisamente na necessidade de historicizar as informações aportadas aos objetos à sua entrada no museu antropológico. Assim, o artigo não aprofunda o estudo das circunstâncias em que os objetos foram obtidos pelos missionários em Angola, mas constitui um ponto de partida crítico para o fazer.

## Coleções “ultramarinas” e património africano: o Museu e Laboratório Antropológico da Universidade de Coimbra entre o colonial e o pós-colonial

Falar hoje em Museu e Laboratório Antropológico da Universidade de Coimbra é evocar uma reconfiguração institucional e disciplinar historicamente situada. Não só o museu antropológico deixou de existir enquanto tal, e passou a ser uma secção do Museu da Ciência da Universidade de Coimbra em 2006, como o ensino e a investigação em antropologia foram integrados num Departamento de Ciências da Vida, criado três anos depois. Com efeito, essas são apenas as mais recentes reconfigurações de uma história que se pode fazer remontar à criação do Museu de História Natural da Universidade pela reforma pombalina de 1772. Desde então, essa história se desdobra em narrativas e práticas que se repercutem na conceção, no uso e na circulação das coleções.

Em 2015, num seminário a propósito dos 130 anos da criação da cadeira de “antropologia, paleontologia humana e arqueologia pré-histórica”, Nélia Dias elencava precisamente os diversos nomes atribuídos ao museu ao longo do tempo e chamava a atenção para a instabilidade das fronteiras disciplinares da antropologia. A criação de tal cadeira, marco da institucionalização da disciplina em Coimbra, fez-se no âmbito da Faculdade de Ciências, trazendo das ciências “naturais” a valorização de um ensino prático e baseado em coleções (Roque, 2010, p.141). Essas incluíam tanto materiais osteológicos como etnográficos que, à medida que as variantes naturalista e culturalista da disciplina se foram articulando na universidade (Santos, 2005, p.78-87), foram ocupando lugares distintos e sendo valorizados de maneiras diferentes. Tais articulações são importantes para compreender, ainda que de maneira necessariamente breve, a história e o estatuto das coleções etnográficas em Coimbra, na transição do colonial para o pós-colonial, contexto no qual a coleção reunida em Angola pelos missionários foi incorporada.

Ao longo da primeira metade do século XX, o programa científico do museu antropológico foi marcado por uma orientação naturalista (Santos, 2005, p.150). Em 1911, foi formalmente criado o Museu e Laboratório Antropológico, que se instalou no Colégio de São Boaventura, tendo sido desenvolvidos alguns esforços de ampliação das coleções e de reorganização do museu (p.150-154). As coleções etnográficas, que tinham sido reforçadas no final do século XIX graças às redes e dinâmicas associadas à expansão da ocupação colonial (Amaral, Martins, Miranda, 2013, p.134-136; Martins, 1985, p.119-129), foram, contudo, relativamente secundarizadas nesse período. Isso voltaria a mudar nos anos 1950, quando surge um plano ambicioso de reorganização do museu, focado nas coleções etnográficas e impulsionado pelas reformas da política colonial e científica da época (Castelo, 2012). Seria então criado na universidade e com ligação à Junta de Investigações do Ultramar um Agrupamento Científico de Estudos Ultramarinos e, dentro deste, uma “secção de Antropologia e Etnologia” (Areia, Rocha, 1985, p.22). Em 1955, a imprensa da Junta publicava o resultado de um processo de inventariação das coleções etnográficas com o título “Catálogo-inventário do Museu de Etnografia do Ultramar do Instituto de Antropologia da Universidade de Coimbra” (Amorim, Morais, 1955). A designação dada ao museu é reveladora da vontade de singularizar as coleções etnográficas, que eram revalorizadas como “ultramarinas” à luz da reforma discursiva e política emergente nos anos 1950 (Castelo, 2014).

Para desenvolver os planos de renovação do museu, o então diretor Alberto Xavier Cunha Marques deslocava-se a Angola, em 1957. Numa entrevista, afirmava: “Nas futuras instalações da Cidade Universitária ficará localizado um amplo Museu de Etnografia do Ultramar” (Marques, 1957, p.128). A visita aos museus em Angola visava comparar coleções, recolher informações e estabelecer futuras colaborações. Nesse sentido, para o Museu do Dundo, o professor de Coimbra levara fotografias de objetos para obter apoio dos conservadores daquele museu no seu estudo e classificação. O relatório do Museu do Dundo, referente a 1957, regista o seu interesse na colaboração desenvolvida na “qualidade de diretor de um Museu Antropológico em formação” ([Museu do Dundo]…, 15 mar. 1958, p.76). Em Luanda, Marques terá certamente visitado o Museu de Angola, que também publicara em 1955 o catálogo da sua coleção etnográfica (Redinha, 1955). No contacto com o Instituto de Angola, Marques (1957, p.219) referia-se à importância de abrir “corrente[s] de intercâmbio” para o Agrupamento Científico, cujo principal objetivo era “aproximar o Ultramar dos universitários”. Essas passagens revelam o esforço que se estava a empreender em torno das coleções etnográficas ditas “ultramarinas” em Coimbra, uma instituição com coleções que remontavam ao século XVIII e que se via como “em formação”. Ao mesmo tempo, a deslocação a Angola sugere que esse esforço não encontrava paralelo nas instituições existentes no país, o que alimentava as ambições de renovação do museu universitário.

A reorganização das coleções, que seriam transferidas para o Colégio de São Bento, na alta universitária, era a principal preocupação do museu (Marques, 1982, p.23), tanto que a viagem a Angola não foi encarada como uma oportunidade para fazer recolhas. Não obstante, a publicação de um “Guia do coleccionador de objetos etnográficos”, por um dos autores do Catálogo-inventário, em 1957, sugere que se tentava também adquirir novas coleções (Amorim, 1957). Fernando Bayolo Pacheco de Amorim apelava aos que viviam com as “populações nativas” e orientava-os na prática de recolher e enviar objetos para o museu. Para além de mencionar o plano de reorganização em curso, Amorim (1957, p.559) assinalava alguma resistência à integração da “etnologia no quadro dos estudos universitários”. Na sua perspectiva, isso devia-se à ideia corrente “de que a etnologia não é mais do que uma recolha de ‘costumes sem uso’, a que se dedicam, para matar o tempo, certas pessoas sem ocupação, porventura um trabalho para reformados do Exército e da Administração”.

Em causa estava um questionamento da cientificidade e utilidade da variante culturalista da disciplina em Coimbra e da sua base museológica. A crítica entendia a etnologia como o estudo de sociedades “primitivas”, cujo desaparecimento resultante do impacto colonial tornava a disciplina obsoleta. Na sua resposta, Amorim (1957, p.559-560) afirmava que a etnologia era tão científica e importante como a “física ou a história”. Ela não só não se tornara obsoleta como revelava a sua utilidade na resolução dos problemas que se faziam sentir nas relações entre “povos colonizadores e colonizados”.^3^ A ancoragem da disciplina no empreendimento colonial não era, em nenhuma das visões, questionada.

Por sua vez, o museu era concebido como um “arquivo de testemunhos”, a partir do qual se poderiam desenvolver estudos etnológicos. No entanto, para que tal fosse possível, como o “Guia” explicava, as recolhas tinham de ser bem organizadas e documentadas. Nas palavras de Amorim (1957, p.561; destaque no original), “o objecto sem o conjunto de informações que lhe dão a ‘qualidade de testemunho’ não tem, para a ciência, o mais pequeno interesse”. Essa preocupação com a “informação” – como garantia de que os objetos poderiam ser tomados como base fiável para o estudo etnológico – refletia a inserção da disciplina e das coleções numa instituição de tradição naturalista, segundo a qual a utilidade científica dos “espécimes” dependia da sua autenticidade, ou seja, da fixação fidedigna da sua autoctonia natural ou cultural, que por sua vez dependia da sua documentação rigorosa (Roque, 2012).

O apelo feito no “Guia” não gerou grandes resultados (Martins, 1985, p.140), ao passo que a reorganização do museu também se mostrava lenta. Seria preciso esperar quase uma década para que, em 1966, se inaugurasse não um “amplo Museu de Etnografia do Ultramar”, mas um modesto “museu didático”, onde as coleções etnográficas tinham sido organizadas segundo critérios geográficos e morfológicos (Areia et al., 1991, p.25; Gouveia, 1983a, p.34). Tal desfecho, certamente, não foi alheio à concretização em paralelo de outro plano de institucionalização da etnomuseologia em Portugal, também respaldado pela Junta de Investigações do Ultramar, que levaria à criação do Museu de Etnologia do Ultramar em Lisboa, em 1965.

Embora as ambições do museu em Coimbra tenham sido frustradas, o caminho trilhado permitiu projetar a variante culturalista e as coleções etnográficas na universidade, influenciando as gerações formadas posteriormente. No ano em que o “museu didático” foi inaugurado, Manuel Laranjeira Rodrigues de Areia terminava a sua licenciatura em ciências biológicas – um membro da Congregação do Espírito Santo, que viria a tornar-se uma figura-chave na articulação entre as duas instituições e no desenvolvimento da antropologia em Coimbra após o fim do regime. A Congregação e os missionários viviam, então, tempos de crise, associada não só aos conflitos em Angola, como ao questionamento mais geral dos propósitos e métodos da missão, patente nas discussões do Concílio Vaticano II (Neiva, 2005, p.511-532). Abandonando a vocação sacerdotal, Areia partiu para Bruxelas para prosseguir a sua formação e começou a dedicar-se ao tema da adivinhação no nordeste de Angola e ao estudo dos cestos de adivinhação dos Cokwe em vários museus europeus (Areia, 1973), partindo para Angola para fazer trabalho de campo nos conturbados anos de 1974 e 1975.

Tudo mudara politicamente, mas a tónica “ultramarina” acentuada nas décadas anteriores continuava a pairar sobre as coleções etnográficas em Coimbra, que provinham, na sua maioria, dos países agora independentes da Guiné-Bissau, Moçambique, Cabo Verde e Angola. Era necessário repensar as coleções, o museu e a antropologia a ele ligada. As dinâmicas que se seguiram nesse sentido podem ser observadas em várias frentes, incluindo uma reorganização das coleções, a atualização dos procedimentos de inventariação, a realização de exposições e a política de aquisições.

**Figura 1:**
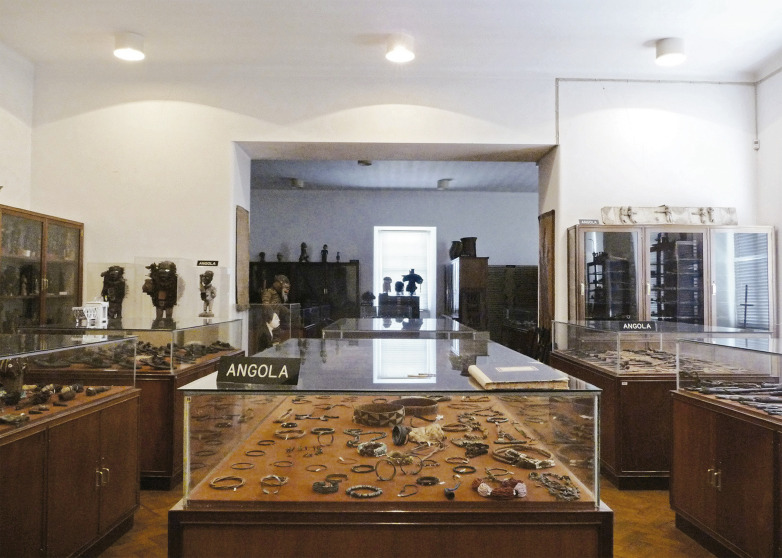
: Antiga reserva visitável da secção de antropologia do Museu da Ciência da Universidade de Coimbra, no Colégio de São Bento (entretanto desmantelada) (acervo da autora)

A ideia de promover a acessibilidade das coleções orientou a sua reorganização em reservas visitáveis (Figura 1) e a atualização das práticas de inventariação (Gouveia, 1980, 1981). Combinando um sistema elaborado de fichas, livros de inventário, arquivo fotográfico e “processos técnicos”, o novo sistema mostra uma renovada importância atribuída ao registo de informações relativas aos contextos de produção e uso dos objetos, bem como à sua “história museográfica” (Gouveia, 1981). O elemento-chave de valorização das coleções no museu – a sua boa documentação – mantinha-se, de alguma forma, o mesmo. Nas palavras do então conservador, Henrique Coutinho Gouveia, “a autenticidade e grau de informação aqui registada influenciarão de forma decisiva o valor documental dos objetos colecionados e, consequentemente, as possibilidades da sua utilização museográfica futura” (Gouveia, 1981 anexo 10).

O objetivo de abrir as coleções e o museu ao público exterior, bem como de “alertar … para o valor cultural de um património que os contactos com territórios africanos têm trazido para Portugal e que muitas vezes tem permanecido anónimo e ignorado”, levou o museu a organizar logo em 1976 a exposição itinerante “Angola: culturas tradicionais” (Gouveia, 1976, p.16, 1983a, p.14). Nos anos seguintes, realizaram-se várias outras exposições, nomeadamente sobre a história da instituição e sobre temáticas associadas à organização das Semanas de Cultura Africana,^4^ dinamizadas pelo recém-criado Centro de Estudos Africanos, cujos objetivos incluíam “contribuir para um novo relacionamento, harmonioso e equilibrado, entre Portugal e os vários países africanos de expressão oficial portuguesa” (Houart, 1985, p.207).

Ao mesmo tempo, desenhou-se uma nova política de aquisições. Por um lado, abriu-se uma linha de investigação associada à constituição de coleções etnográficas portuguesas (Gouveia, 1979a, 1979b), que seguia o impulso da geração de antropólogos sociais portugueses que, depois do 25 de abril, se dedicou a “redescobrir uma certa portugalidade” (Branco, 2014; Pina Cabral, Macagno, 2001, p.354-355). Por outro lado, uma segunda linha enquadrou-se numa reflexão sobre o estatuto das coleções “ultramarinas” e do papel do museu na situação pós-colonial e pautou-se por um mapeamento das coleções privadas existentes no país (Gouveia, 1978, p.14; Martins, 1985, p.142).

A esse respeito, um dos efeitos da revolução de abril foi, segundo Areia, a “imediata revalorização e acentuada procura, sobretudo por parte de colecionadores estrangeiros, do património africano existente em Portugal” (Areia, 1986, p.141; Gouveia, 1983b, p.17-19). Como tal, surgira um mercado ilegal para esses objetos, tendo-se mesmo registado roubos em instituições museais como o Museu Nacional de Arqueologia e Etnologia, de onde teria desaparecido mais de 1/4 da coleção africana (Areia, 1986, p.142; Gouveia, 1983b, p.49).^5^ Face ao problema, o museu universitário de Coimbra procurava reclamar para si um papel na salvaguarda e valorização de tais coleções.^6^ Esse papel era tão mais reforçado quanto se constatava uma “crise dos museus da especialidade” e um desinteresse por parte dos organismos estatais centrais, que tinha como consequência a saída descontrolada desses bens do país (Gouveia, 1983b, p.18). A crítica dirigia-se em parte ao Museu de Etnologia do Ultramar, cuja situação institucional se complicara com a extinção do Ministério do Ultramar e dos organismos dele dependentes (Gouveia, 1997, p.281-358).

Manuel Laranjeira Rodrigues de Areia e Henrique Coutinho Gouveia foram, sem dúvida, os principais impulsionadores da reflexão sobre a situação das coleções etnográficas reunidas em contexto colonial existentes em Portugal após 1974-1975, agora vistas como “património africano” cuja valorização tinha de passar pela criação de novas relações com os países independentes e pela adoção de uma nova política patrimonial. No opúsculo *As colecções etnológicas de origem ultramarina no contexto de uma política do património cultural*, Gouveia (1983b, p.63) afirmava que se tratava de uma preocupação partilhada entre os “principais museus de etnologia portugueses e a que o Museu e Laboratório Antropológico da Universidade de Coimbra já vinha dedicando atenção desde 1974”. O conservador fazia várias recomendações, entre as quais a realização de um levantamento “das coleções africanas na posse de colecionadores ou de instituições particulares” (Gouveia, 1983b, p.26). O museu forneceria aos colecionadores “apoio técnico e científico na classificação dos objetos”, bem como serviços de conservação e restauro, em troca de uma cláusula preferencial na sua eventual aquisição. O panorama traçado pelos antropólogos do museu universitário era tão realista e a sua política de aquisições foi tão bem-sucedida que, entre a segunda metade da década de 1970 e o início da década de 1980, o museu registou um crescimento significativo do seu acervo, em resultado da aquisição de várias coleções privadas, sobretudo de Angola (Areia, 2009, p.27; Martins, 1985, p.142).

Foi nesse contexto, marcado pelo dealbar de uma reflexão crítica sobre o estatuto das coleções coloniais nos museus portugueses e sobre o papel destes depois do 25 de abril e das independências, que a coleção reunida pelos missionários em Angola veio a ser incorporada no museu antropológico, onde se encontra em depósito até hoje.

## O estudo da coleção e da sua circulação: passos dados em direção a novas questões

A ideia de que os objetos no museu não possuem valores e significados intrínsecos, e que uma coleção não é uma entidade coesa e estática, mas o produto de uma circulação histórica e epistémica em contínuo andamento que tem de ser desembrulhada, constituiu o ponto de partida do estudo da coleção. Assim, o primeiro passo deste estudo foi examinar o processo de incorporação e compreender as práticas de catalogação realizadas no museu, tendo em conta a sua contextualização histórica discutida acima. As principais questões que foram guiando a pesquisa prenderam-se com o modo como os objetos saíram do museu missionário, o que foi considerado importante registar nesse processo e as razões subjacentes a essa decisão.

O estudo do processo de incorporação mostrou desde logo a situação em que se encontrava o museu missionário. Em meados de 1978, o superior provincial da Congregação do Espírito Santo em Portugal descrevia-o nos seguintes termos: “Os objetos que temos no Museu de Carcavelos não estão estimados nem aproveitados; apenas se fez um trabalho sobre os testos de Cabinda. Mas penso que a Congregação não se deve desfazer desses objetos” (Oliveira, 7 jul. 1978). Os missionários pareciam já não saber o que fazer com a coleção. Como muitos dos seus congéneres pela Europa, que foram fechando à medida que as organizações missionárias se foram reestruturando a partir da década de 1960 (Wingfield, 2017, p.232), o museu criado pelos espiritanos portugueses no seminário maior da Torre d’Aguilha, em Carcavelos, parecia ter não só perdido a sua relevância, como se tinha tornado um fardo. Reconhecia-se que, para manter um tal museu, seria necessário “estimar” e estudar os objetos. No passado, alguns missionários tinham-se dedicado a esse estudo, como era o caso dos “testos de Cabinda” (Martins, 1968; Vaz, 1969), mas agora já não se viam na posição de o fazer. Ainda assim, a Congregação desejava manter a propriedade da coleção.

O que se seguiu depois foi um processo longo e faseado, com implicações diversas, do qual se podem destacar alguns aspetos. O primeiro prende-se com a questão do mercado dito de “arte tribal” e do incremento da circulação ilícita de objetos africanos devido à instabilidade política em Portugal e nos países agora independentes, que constituía, como se viu, uma das principais preocupações dos antropólogos do museu universitário. Este assumia-se então como uma instituição com capacidade e responsabilidade para responder ao problema, o que não era visto como contraditório com o estabelecimento de relações e a compra de objetos em antiquários, algo que também se vinha fazendo no âmbito da política de aquisição de coleções. Foi graças a essas relações que os antropólogos souberam, meses depois da carta do provincial espiritano, que um conhecido antiquário e leiloeiro de Lisboa fizera menção à coleção missionária e aos “testos de Cabinda”, o que despertara o interesse de particulares na Bélgica (Lino, 15 fev. 1979). Para evitar que a coleção saísse de Portugal, os universitários enviaram de imediato uma proposta à Congregação, indo ao encontro do que os missionários desejavam (Gouveia, 22 fev. 1979). Estes manteriam a propriedade da coleção e seriam feitos depósitos temporários por lotes, permitindo que os objetos fossem estudados, classificados, conservados e fotografados, segundo o modelo concebido por Gouveia para o mapeamento das coleções privadas. Referia-se também que se planeava uma nova exposição sobre Angola, mostrando que as coleções eram bem “aproveitadas” em Coimbra.^7^ A proposta foi aceite pelos missionários, o que nos leva ao segundo aspeto a destacar, que diz respeito à necessidade de desagregar o que a coleção é hoje em diferentes fluxos de coleções.

Em abril de 1979, eram registadas 42 entradas nos Livros de Depósito do museu antropológico, em resultado de uma primeira seleção efetuada por Gouveia no seminário. Contrariamente ao planeado, o restante acervo missionário não seria transferido por lotes, mas na sua totalidade para Coimbra, resultando no registo de 844 entradas nos Livros de Depósito em 1984, passando a coleção a totalizar 886 registos. Como adiante se verá, este número tem subjacente uma série de operações com efeitos a considerar. Por fim, quase duas décadas depois, em 2001, os Livros registam um novo depósito associado à Congregação do Espírito Santo. Os missionários tinham, entretanto, encontrado outro núcleo de objetos angolanos que não pertenceu ao museu da Torre d’Aguilha, motivo pelo qual não tinha integrado o depósito anterior. Tratava-se de uma segunda coleção de “testos de Cabinda”, cuja história não era perfeitamente conhecida. Dois dos missionários que se dedicaram ao tema – os padres José Martins Vaz, visto como responsável pela coleção que já se encontrava em Coimbra (composta por 274 registos dos referidos “testos”, hoje designados “tampas de panela”), e Joaquim Martins – tinham sido consultados, mas sem que se chegasse a uma conclusão definitiva. Não obstante, pensava-se que o conjunto correspondia às recolhas feitas pelo padre Joaquim Martins, entre 1941 e 1949, durante o seu primeiro período de trabalho na missão do Lucula, situada na margem esquerda do rio homónimo, próxima da fronteira com o então Congo Belga, tendo o missionário trazido os objetos para Portugal aquando da sua primeira licença em 1949 (Moreira, 18 nov. 2001). As recolhas feitas por Vaz eram posteriores, e os objetos tinham sido estudados por ele no museu missionário depois do seu regresso a Portugal, em 1958. Assim, em 2001, eram feitos 67 novos registos nos Livros de Depósito, passando a coleção associada aos Missionários do Espírito Santo em Coimbra a totalizar 953 registos.

O terceiro aspeto a salientar prende-se com a sequência de acontecimentos entre o primeiro e segundo depósito e com as consequências daí resultantes, tanto no que respeita aos destinos dos objetos então constantes do museu missionário como às possibilidades da sua pesquisa hoje.^8^ Como se viu anteriormente, a seleção feita por Gouveia em 1979 resultou em 42 entradas registadas no Livro de Depósitos, sendo a cada objeto atribuído um número e um nome, aos quais se acrescentou a sua descrição morfológica. A análise desses nomes mostra um esforço de categorização com base em critérios morfológicos e funcionais, que configura uma operação específica de acrescentar informação aos objetos considerada útil no seu novo contexto museal. Assim temos, “testo de panela” (6), “escultura” (8), “estatueta” (7), “caixa para remédios” (1), “objeto de fecundidade” (2), “caurins de fecundidade” (1), “máscara” (1), “cesto de adivinho” (1), “instrumento musical” (1), “instrumento musical de arco” (2), “chocalho” (4), “chocalho com badalo de ferro” (1), “puíta” (1), “bilha” (1), “cesto” (1), “cabacita” (1), “ventosa” (1), “chifre” (1) e “pulseira” (1) (MLAUC, s.d.). Os dois critérios que mais influenciavam a valorização dos objetos na perspetiva do conservador, como acima se notou, eram sua “autenticidade e seu grau de informação”. O registo dos objetos em 1979 mostra como ambos se podiam articular. Alguns nomes com os quais os objetos foram batizados pelos antropólogos apontam para sua valorização como “documentos” para o estudo de temas como a fecundidade ou a adivinhação, mas a maioria é demasiado geral e sugere que o conservador terá baseado a sua seleção na “autenticidade” dos objetos, patenteada por meio das marcas de uso e da ligação dos missionários com o terreno. Com efeito, o mais notório desse primeiro registo é a confiança absoluta na capacidade de os objetos sozinhos serem tomados como base para o estudo de temáticas então consideradas de interesse etnológico. Nenhuma menção é feita a informações existentes no museu missionário ou fornecidas pelos missionários.

É precisamente nesse sentido que se pode entender a avaliação do acervo missionário elaborada por Areia aquando do segundo depósito em 1984, focada em quatro pontos principais:

- A heterogeneidade dos objetos e o seu valor escasso em termos de peças ornamentais, não obstante o valor indiscutível de algumas peças como objetos etnográficos.
- A diversidade dos objetos no que diz respeito à qualidade, havendo alguns muito bons, outros de valor duvidoso e outros provavelmente sem interesse etnográfico.
- A situação precária da coleção em termos de insegurança, aliás comprovada já pelo desaparecimento de algumas peças nomeadamente de marfim.
- A capacidade das instalações do Museu e Laboratório Antropológico, que, além da galeria de exposição permanente que conta abrir brevemente ao público, tem amplas reservas visitáveis (onde já está parte da coleção de Carcavelos) e outras reservas (no sótão) com o mínimo de condições de preservação (MLAUC, 8 jun. 1984).

A principal característica do acervo missionário era, para o professor de antropologia, a discrepância de valor dos objetos. Por um lado, havia “objetos etnográficos” vistos como “muito bons” ou mesmo de “valor indiscutível”, mas havia também, por outro lado, “peças ornamentais” de “valor escasso” ou “duvidoso”. Tal oposição mostra como os objetos foram investidos de valor pelo museu antropológico, valor esse que residia no seu potencial “documental” para o estudo de certos temas, e não de outros. As “peças ornamentais”, como as que teriam sido produzidas para o mercado ou no contexto das missões, não tinham “interesse etnográfico”. Ou melhor, aquilo que elas permitiam documentar não era então considerado relevante para a antropologia.

Não obstante, o museu antropológico dispôs-se a receber a totalidade do acervo missionário. No museu, as “peças de valor indiscutível” seriam apartadas e expostas, ao passo que as restantes iriam para as reservas não visitáveis. A triagem passaria também por um critério disciplinar, pelo qual os “troféus de caça” seriam transferidos para o Museu e Laboratório Zoológico da universidade, onde passariam a ser “espécimes zoológicos”. Esses critérios reforçavam o entendimento do “objeto etnográfico” como aquele que documentava um meio cultural abstraído da situação missionária e colonial, ao passo que a inserção da antropologia no contexto de um museu de história natural resultava numa apreensão mutuamente excludente das duas categorias – objeto-cultural e objeto-animal – cujo mínimo denominador comum, o missionário (caçador ou etnógrafo), permanecia, em ambos os casos, irrelevante. Por fim, o parecer sublinhava o papel do museu antropológico na “preservação de um elemento do património cultural importante”, cabendo aos missionários a prestação de “um relevante serviço à comunidade colocando a coleção em condições de melhor segurança e preservação, facilitando o acesso aos estudiosos e a sua consequente divulgação” (MLAUC, 8 jun. 1984).

**Figura 2:**
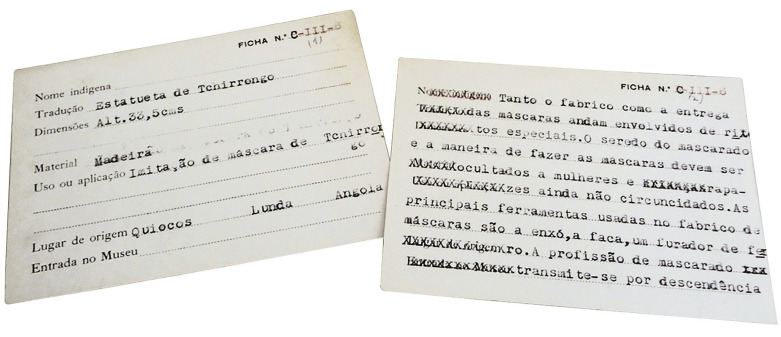
: Exemplo de uma ficha de inventário do museu missionário da Torre d’Aguilha (acervo da autora)

A aceitação da proposta pela congregação, em meados de 1984, abriu uma nova etapa na circulação da coleção. O processo foi acompanhado pela nova conservadora das coleções em Coimbra, Maria do Rosário Martins, e, ao contrário do que se passara em 1979, não só todo o acervo missionário foi transportado para o museu universitário como também toda a papelada que lhe estava associada, cujo potencial documental foi então reconhecido pela conservadora. Assim, os objetos musealizados pelos missionários, mesmo aqueles que iriam parar aos cantos mais escondidos das reservas antropológicas, puderam continuar a sua trajetória museal e patrimonial, o que, por sua vez, possibilitou o seu estudo atual. Ao mesmo tempo, a atenção dada pela nova conservadora a todo um conjunto de papéis, fichas, rótulos, pequenas notas, dísticos e etiquetas existentes no museu missionário (Figura 2), fazendo-o circular a par dos objetos para Coimbra, fornece uma oportunidade única para examinar as várias camadas resultantes das mais diversas práticas de produção de saberes e de “catalogação da cultura” (Turner, 2020) desenvolvidas em torno dos objetos.

Uma parte desse trabalho torna-se visível por meio de um ficheiro composto por uma caixa de madeira retangular, com vários separadores, que hoje contém 339 fichas cartonadas. Tal ficheiro evidencia um esforço missionário de inventariação que assentava na numeração e etiquetagem dos objetos, e na criação de fichas correspondentes onde se podia escrever informação numa série de campos, nomeadamente “Nome indígena”, “Tradução”, “Uso ou aplicação”, “Lugar de origem”, “Entrada no Museu” e “Particularidades” (no verso). Esse método foi adotado pelos missionários em 1960, graças à passagem de um dos etnólogos do Museu do Dundo pelo seminário, pelo que reflecte as perspetivas e práticas etno-museológicas desse museu (não é possível aprofundar a sua análise aqui; ver Amaral, 2018).

**Figura 3:**
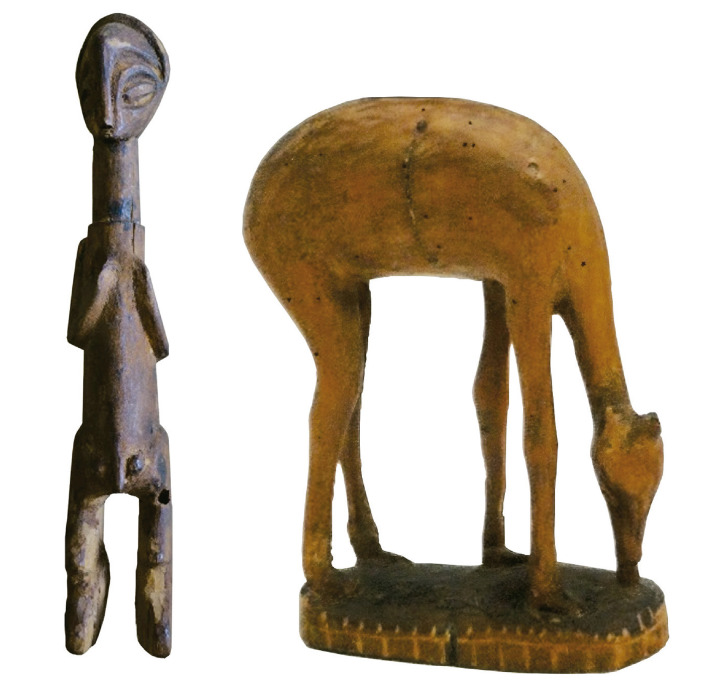
: Os objetos n.ANT.D.84.1.857 e ANT.D.84.1.649 (acervo da autora)

Durante o estudo da coleção, procurou-se compreender como esse sistema se desenvolveu e foi aplicado, bem como as informações lá registadas e o que elas permitem saber sobre a maneira como os objetos tinham sido obtidos pelos missionários em Angola e como teriam circulado entretanto. O seu preenchimento revelou-se bastante inconsistente, mas ainda assim o ficheiro contém várias pistas de pesquisa. Essas apontam para uma quantidade significativa de objetos que entraram no museu missionário entre 1954 e 1964, sobretudo de regiões como as atuais províncias de Cabinda, Zaire, Uíge, Bengo, Malange e Lundas, com alguns registos também associados à área da Huíla. O ficheiro contém também os nomes de alguns missionários associados à circulação dos objetos, ainda que, ao contrário do que seria de esperar, sejam escassas as referências às missões. Essas aparecem em algumas notas e etiquetas, como os exemplos incluídos na figura acima: uma nota num pedaço de papel timbrado do bispado de Daniel Gomes Junqueira (Nova Lisboa, atual Huambo, entre 1941 e 1970) com a inscrição “objecto feito pelas internas da Missão de Caconda”, o que aponta para o facto de objetos produzidos no contexto das missões, inclusivamente das missões femininas, fazerem parte da coleção (infelizmente não foi possível identificar o objeto em questão); e ainda duas etiquetas em papel, numa das quais se pode ler “‘Ídolo’ dos caçadores Lunda – quiocos Ganguelas e outros”, e noutra onde se escreveu “Missão do Mussolo/Um Bambi” (Figura 3).

Trata-se de dois objetos que estariam em lados opostos do espectro de valor etnográfico segundo o entendimento expressado aquando das primeiras abordagens dos antropólogos à coleção missionária discutida acima. Por um lado, uma escultura em madeira com sinais de uso e que é, por isso, capaz de “testemunhar” esse uso. A etiqueta missionária acrescenta informações que reforçam essa mesma valorização, nomeadamente uma terminologia, entretanto substituída – “ídolo” –, um contexto de uso e uma série de atribuições étnicas. Por outro lado, uma escultura zoomorfa em madeira, sem nenhum outro elemento aparentemente de interesse etnográfico, portanto, uma “peça decorativa”. No entanto, a perspetiva adotada durante o estudo da coleção procurou problematizar esse entendimento. A compreensão desses objetos no contexto da sua circulação missionária permanece fundamental para a discussão crítica das suas histórias, aberta a considerar o modo como a evangelização católica foi vivida em Angola e os seus mais variados impactos e legados, nomeadamente no que respeita ao desenvolvimento e à apreciação do artesanato e das artes populares. Nesse espírito, uma análise mais atenta da segunda etiqueta revela, por exemplo, que a aparentemente trivial atribuição do termo “Bambi” ao objeto reflecte a origem etimológica deste na palavra *mbámbi* em Kimbundu, língua falada na missão do Mussolo, também referida na etiqueta, que foi criada em 1937 e ainda existe atualmente, pertencendo à Arquidiocese de Malange. Terá este “Bambi” sido comprado por algum dos missionários trabalhando no Mussolo? Terá sido oferecido? Quem terá sido o seu criador? Seria a oficina onde ele foi produzido próxima da missão?

Igualmente, a remoção do termo “ídolo” e a atribuição da designação “escultura” colocam desafios importantes à pesquisa das coleções associadas à atividade missionária em contexto colonial, que não será possível discutir aqui a fundo (ver, a esse propósito, Hersak, 2022). Cabe apenas sublinhar a possibilidade de religar a etiqueta ao objeto e, desse modo, examinar essa camada da sua circulação histórica e epistémica, o que acontece com diversos outros objetos da coleção, designados no ficheiro missionário como “feitiços”. Porquanto o termo “ídolo” não seja atualmente aceitável nas práticas de catalogação museal, uma vez que ele revela um discurso missionário depreciativo dos objetos e práticas africanas de culto não católico, a sua persistência no arquivo do museu permite problematizar a sua relação metonímica com a recolha missionária e situá-lo historicamente ou pelo menos levantar questões específicas nesse sentido. Por exemplo: o que terá determinado a trajetória do objeto até ele chegar às mãos missionárias? Terá o caçador, ou um conjunto de caçadores das regiões em Angola onde aqueles etnónimos circulavam, renunciado ao seu uso por influência do missionário ou terá sofrido consequências da remoção forçada (pelo missionário? Por um catequista?) do dito “ídolo” na sua atividade?

Outro passo no estudo da coleção beneficiando de uma análise atenta à produção antropológica e missionária de saberes e narrativas em torno dos objetos pode ainda ser aqui mencionado. Como se afirmou anteriormente, a coleção que constava no museu missionário é hoje composta por 886 registos inscritos nos Livros de Depósito em 1979 e 1984, aos quais se podem acrescentar 67 feitos em 2001, referentes a um segundo núcleo de objetos associados às recolhas de um missionário da Congregação. Durante o estudo, identificou-se ainda outro registo inventariado no Livro de Tombo (ANT.98.4.1), que corresponde a um objeto que comporta uma etiqueta com numeração do museu missionário, que ainda conserva a respetiva ficha. O levantamento e a observação dos objetos, desenvolvidos exaustivamente graças ao interesse e abertura da então conservadora das coleções, levaram também à realização de sete novos registos. Seis referentes a objetos de pequena dimensão, que se encontravam dentro de cestos de adivinhação, sendo, por isso, considerados componentes desses mesmos cestos, mas que possuíam etiquetas de inventariação missionária que permitiram religá-los às informações constantes nas fichas e, assim, autonomizá-los. E, finalmente, um sétimo, respeitante a uma representação figurativa em madeira de um missionário a pregar numa aldeia e, por isso, encontrado num dos cantos recônditos das reservas. A inventariação individualizada desses objetos confere-lhes um novo estatuto e uma nova visibilidade. A coleção passa a totalizar 961 registos numerados. Esse aspeto final sublinha a ideia de que as coleções não são entidades estáticas e coesas, e que as práticas de catalogação têm uma historicidade própria, em mutação, que comporta efeitos sobre os objetos e sobre os seus possíveis destinos.

## Considerações finais

Não sendo uma novidade, nem nos meios museais nem académicos, o estudo das coleções que hoje se encontram nos museus, em particular daquelas que se constituíram durante o colonialismo ou em relação com as dinâmicas da dominação colonial, procura responder a questões e segue métodos que têm, de alguma forma, variado. A persistência da tónica no “valor documental” dos objetos em museus, como o Museu e Laboratório Antropológico da Universidade de Coimbra, mostra o interesse histórico em tomar as coleções como base de estudo. Contudo, se anteriormente o foco se colocava na “autenticidade” dos objetos e na sua vida cultural abstraída das circunstâncias coloniais e missionárias que determinaram a sua circulação até o museu, hoje são estas mesmas circunstâncias – o tipo de transações que implicaram, quem tomou parte nelas – que constituem o foco de interesse, aquilo que se pretende que os objetos contribuam para “documentar”, seja no seu estudo, seja nas dinâmicas de exposição e de gestão museal.

A problematização fundamental sobre o que constitui uma coleção, ou melhor, sobre como uma coleção se tornou naquilo que ela é quando nos dispomos a estudá-la, é um passo crítico inicial no estudo das coleções. Que operações e critérios lhe conferem unidade e valor ao longo do tempo? O que fica de fora desse processo? O que ganhamos ao analisar o processo museal de fazer coleções ou aquilo a que os autores de um livro recente chamaram a “fábrica das coleções” (Van Beurden, Gondola, Lacaille, 2023b)? Perguntar sobre como determinada coleção se tornou aquilo que é no momento atual implica não tomar como garantido: (1) a coleção como unidade coesa e estável; (2) os valores e significados atribuídos aos objetos; (3) a sua circulação histórica e epistémica, ou seja, a circulação interligada e contingente de objetos e narrativas, e o modo como essa circulação produz efeitos sobre os significados e valores dos objetos. Implica, assim, também não tomar por garantido o seu futuro.

As narrativas e informações que rodeiam as coleções nos museus são o produto de práticas historicamente contingentes, seguindo critérios que necessariamente enfatizam certas perspetivas, classificações, terminologias e tipos de informação, e excluem outros. A compreensão crítica do que podemos ou não podemos encontrar no museu e no arquivo, a nossa capacidade de historicizar as práticas de “catalogação da cultura” e de produção de informação em torno dos objetos, é outro passo fundamental no estudo das coleções. A partir daqui, torna-se possível compreender as suas limitações e os seus enviesamentos e encontrar métodos que nos permitam recuperar o que hoje vemos como relevante e ausente desse trabalho.

O presente artigo concentrou-se no estudo de uma coleção associada às atividades dos Missionários do Espírito Santo, em Angola, durante o período colonial, hoje depositada na secção de antropologia do Museu da Ciência da Universidade de Coimbra. A abordagem adotada permitiu perceber que ela resultou de vários fluxos ocorridos entre 1979 e 2001, contabilizando hoje 961 registos, que englobam o acervo de um antigo museu missionário e um conjunto de objetos associados às recolhas feitas por um padre espiritano na região de Cabinda, trazidos por ele para Portugal, mas não integrados no dito museu missionário.

Ao desmembrar o sentido de unidade da coleção, a análise aprofundou a circulação histórica e epistémica dos objetos, com confluências, divergências e implicações distintas. Ao mesmo tempo, ela mostrou como valores e significados foram sendo atribuídos aos objetos, tendo em conta os contextos institucionais subjacentes à sua circulação. Assim, começou-se por discutir uma parte da história do museu antropológico de Coimbra na transição do período colonial para o pós-colonial, o que permitiu dar conta do dealbar de uma reflexão sobre o estatuto das coleções coloniais em Portugal, precursor dos debates atuais sobre os legados coloniais nos museus. Tal reflexão tinha por base uma preocupação com o tráfico ilícito de objetos africanos e a sua revalorização como património africano, e passou por estratégias que visavam melhorar a visibilidade e acessibilidade da coleção e pelo apelo a novas formas de cooperação com os países independentes de onde as coleções provinham. Ainda que sem colocar explicitamente a questão da restituição, essas estratégias encontram ecos nas que têm vindo a ser desenvolvidas mais recentemente e nos debates que têm mobilizado os projetos de “pesquisa de proveniência”.

Os objetos da coleção foram atravessando processos de significação e valorização, associados aos discursos e práticas da missão, do estudo antropológico e da patrimonialização. A conclusão de que todo o acervo do museu missionário e as respetivas narrativas foram incorporados em Coimbra foi crucial e abre novas possibilidades de pesquisa, assentes na recuperação das ligações missionárias entre histórias e objetos, tipicamente negligenciadas pelo filtro da “autenticidade” colocado à entrada dos museus antropológicos. Torna-se, assim, possível trazer a missão de volta à coleção, o que, ao contrário do que possa parecer, não constitui um reforço das dinâmicas coloniais de poder que condicionaram e ainda condicionam, de diversas maneiras, a circulação da coleção e o seu estudo atual. Constituindo a missão um fenómeno complexo, que não pode ser reduzido às ações e perspetivas dos missionários, a proposta de trazer a missão de volta à história da coleção abre, antes, a possibilidade de “restituir a coevidade” (Ivanov, 2017) dos interlocutores e dos emaranhados sociais e materiais que determinaram a sua circulação. Assim, pretende-se contrapor a marginalização antropológica do fenómeno missionário e do catolicismo das populações africanas (Robbins, 2007). Tal marginalização teve como corolário nos museus o descarte das histórias missionárias e o enfoque em objetos vistos como “tradicionais”, em detrimento das manifestações materiais da situação missionária e colonial, que as coleções reunidas pelos missionários têm o potencial de problematizar.

Ecos dessa marginalização ressoam nos debates atuais sobre as coleções coloniais nos museus e sobre os seus futuros. Quando a questão da religião é abordada, o foco de interesse é, quase invariavelmente, colocado no impacto destrutivo da atividade missionária e na “recuperação” de perspetivas e práticas ditas “indígenas” e “tradicionais”, em detrimento de perspetivas e práticas que igualmente se poderiam dizer “indígenas”, mas também cristãs. Quando projetos colaborativos são desenvolvidos e partes interessadas são envolvidas, é mais provável que especialistas ou praticantes religiosos entendidos como “indígenas” ou “tradicionais” sejam convidados aos museus do que padres católicos.

A atenção às práticas missionárias de coleção, criticamente consciente dos problemas e enviesamentos dos arquivos coloniais e missionários, permite abordar novas histórias em torno das coleções e discutir o seu estatuto patrimonial à luz dos legados do colonialismo, mas também do catolicismo. Colocar a discussão nesses termos implica refletir sobre o carácter emaranhado e negociado dos legados (L’Estoile, 2008) e levantar questões difíceis, como: a quem pertencem esses objetos, ou como podemos equacionar criticamente o estatuto da coleção hoje em Coimbra? Como legado antropológico? Como património português ou património angolano em Portugal? Como património da Congregação do Espírito Santo? O que é a Congregação do Espírito Santo hoje, em Portugal e em Angola? O que poderão significar estes objetos e as suas histórias para as pessoas que vivem em contextos marcados pela sua circulação e pelo catolicismo em Angola e suas diásporas? Em suma, as coleções nos museus, hoje, podem também ser tomadas como um prisma de análise sobre a maneira como as pessoas lidaram com a evangelização católica e seus efeitos, no passado e no presente. O seu estudo abre novas possibilidades para entender a complexidade das histórias de circulação dos objetos e os legados da missão e do colonialismo nos museus.

## Data Availability

Não estão em repositório.

## NOTAS

1 Note-se que, cinco anos antes, o governo alemão já disponibilizara um fundo de 1,9 milhões de euros para a investigação sobre as colecções da era colonial nos museus do país com vista a provar que certos objetos de África, Ásia, Austrália e Pacífico tinham sido pilhados (Brown, 5 fev. 2019).

2 Sobre a mais recente abordagem expositiva desenvolvida no Museu da Ciência da Universidade de Coimbra, a partir de uma reinterpretação do modelo dos gabinetes de curiosidades, ver o artigo publicado nesta revista por João Brigola e Paulo Trincão (2023).

3 Amorim formara-se em ciências histórico-filosóficas na Faculdade de Letras da Universidade de Coimbra. Como notado por outros autores, defendia uma visão integracionista do Império, ancorada no campo da direita monárquica coimbrã, que era, apesar das ligações institucionais estabelecidas, crítica de Adriano Moreira e mais tarde da política marcelista (Marchi, 2008, p.572).

4 Na primeira semana, em 1982, foi reposta a exposição “Angola: culturas tradicionais”. A segunda, contou com a exposição “Angola: símbolos do poder na sociedade tradicional” (1983), e, na terceira semana, a exposição foi dedicada ao tema “África: instrumentos musicais” (1984). Todas essas exposições foram acompanhadas pela publicação de catálogos.

5 Em 1986, Gouveia refere-se concretamente ao roubo de uma figura Tchibinda Ilunga do Museu Nacional de Arqueologia e Etnologia (atual Museu Nacional de Arqueologia), que teria, entretanto, sido recuperada por intervenção de Marie-Louise Bastin – que relata parte do caso num artigo publicado na revista *African Arts* (ver Bastin, 1976) – e ainda ao “desaparecimento de mais de 25% da coleção africana deste Museu” no início de 1976, informação baseada num “relatório”, possivelmente um relatório do próprio museu a que o autor teve acesso, mas cuja referência não fornece. É possível que se trate do artigo “Actividades do Museu Nacional de Arqueologia e Etnologia: relatório sobre a secção colonial do Museu Nacional de Arqueologia e Etnologia”, de 22 de Janeiro de 1976, publicado na revista *O Arqueólogo Português*. Os números desse periódico estão disponíveis em versão digital na página do atual Museu Nacional de Arqueologia.

6 Note-se que esta reflexão acompanha a assinatura, em 1970, da Convenção da Unesco sobre as “Medidas a adoptar para proibir e prevenir a importação, a exportação e a transferência ilícitas de propriedade de bens culturais”. Ver texto integral em Convention... (14 nov. 1970).

7 Trata-se da exposição “Angola: culturas tradicionais”, realizada em 1982.

8 Por limitações de espaço, a presente análise concentra-se na coleção correspondente ao museu missionário e não abrange a coleção associada ao padre Joaquim Martins (ver Amaral, 2018).
